# Posterior Shoulder Instability in Contact Athletes: Recognition, Evaluation, and Management

**DOI:** 10.7759/cureus.107706

**Published:** 2026-04-25

**Authors:** Jaxon S Boley, Sean Rockwell, Jason Hunt

**Affiliations:** 1 Orthopedic Surgery, American University of the Caribbean, Miramar, USA; 2 Orthopedic Surgery, Ross University School of Medicine, Miramar, USA; 3 Orthopedic Surgery, The Joint Preservation Center, Lexington, USA

**Keywords:** arthroscopic stabilization, contact athlete, glenoid bone loss, posterior labral tear, posterior shoulder instability, return-to-play

## Abstract

Posterior shoulder instability is an important cause of shoulder pain and dysfunction in contact and collision athletes, but it is often underrecognized because many patients present with vague posterior shoulder pain and activity-related symptoms rather than a clear dislocation event. Repetitive loading in flexion, adduction, and internal rotation, common during blocking, tackling, and other push-based activities, can stress the posterior capsulolabral complex and produce a spectrum of pathology ranging from pain-predominant microinstability to recurrent symptomatic instability. Accurate diagnosis depends on careful pattern recognition through history, physical examination, and imaging interpreted in the proper clinical context. Nonoperative management with structured rehabilitation remains an appropriate initial strategy for selected athletes, although this approach may be less predictable in high-demand collision sports. Operative treatment is often considered in athletes with persistent symptoms, recurrent instability, or structural pathology that reduces the likelihood of success with rehabilitation alone. Osseous morphology, including posterior glenoid bone loss, dysplasia, and version abnormalities, may also influence treatment selection in selected cases. Return-to-play decisions should be criterion-based rather than time-based and should emphasize restoration of pain-free function, near-symmetric strength, dynamic stability, psychological readiness, and tolerance of sport-specific loading. Current evidence supports an individualized approach to management, but further athlete-specific prospective studies are needed to refine morphologic risk stratification, treatment selection, and return-to-play decision-making.

## Introduction and background

Posterior shoulder instability is an important but frequently underrecognized source of shoulder pain and dysfunction in athletes who participate in contact and collision sports. In this review, the term contact and collision athletes is used to describe sports in which repetitive player-to-player or player-to-surface impact commonly exposes the shoulder to high loads, including football, rugby, and wrestling. Compared with anterior instability, posterior instability more often presents without a clear dislocation event and instead manifests with posterior shoulder pain, weakness, mechanical symptoms, or a subjective sense of shifting under load [1,2]. This difference in presentation may delay recognition and treatment.

Posterior instability represents a minority of glenohumeral instability cases overall, but it appears particularly relevant in athletes exposed to repetitive posteriorly directed axial loading [1,2]. In contact and collision sports, these loads are commonly encountered during blocking, tackling, and other push-based activities, especially when the arm is positioned in flexion, adduction, and internal rotation [2,3]. Repetitive exposure to this mechanism can contribute to capsulolabral injury, symptomatic microinstability, and recurrent posterior translation.

Posterior shoulder instability is best understood as a continuum rather than a single uniform entity. Some athletes present with pain-predominant microinstability and subtle functional limitation, whereas others develop recurrent symptomatic subluxation or, less commonly, frank posterior dislocation [1-3]. Because the clinical presentation, imaging findings, treatment options, and return-to-play implications differ across this spectrum, a focused review of posterior instability in contact and collision athletes is clinically relevant.

This narrative review is intended for clinicians caring for athletes with suspected posterior shoulder instability and focuses on recognition, diagnostic evaluation, treatment strategies, and return-to-play considerations. The following sections summarize the current literature on epidemiology, mechanisms of injury, pathoanatomy, clinical presentation, physical examination, imaging evaluation, nonoperative and operative management, and current controversies.

## Review

Methods

This article is a narrative review of the literature on posterior shoulder instability in contact and collision athletes, with an emphasis on epidemiology, mechanisms of injury, pathoanatomy, clinical presentation, diagnostic evaluation, treatment strategies, and return-to-play considerations. A literature search was performed using PubMed/MEDLINE and Google Scholar for English-language studies published from January 2002 to April 2026. Search terms included combinations of “posterior shoulder instability”, “posterior glenohumeral instability”, “contact athletes”, “collision athletes”, “football”, “rugby”, “wrestling”, “Kim test”, “jerk test”, “glenoid bone loss”, “labral repair”, “rehabilitation”, and “return to play.” 

Eligible studies included original clinical studies, cohort and comparative studies, biomechanical investigations, systematic reviews, meta-analyses, consensus statements, diagnostic studies, and review articles relevant to posterior shoulder instability. When available, priority was given to athlete-focused and sport-specific literature, along with more recent and higher-level evidence. Foundational older studies were retained when central to the understanding of posterior instability. Studies were excluded or deprioritized if they were non-English publications, conference abstracts without full text, isolated case reports, or not directly relevant to posterior shoulder instability. Studies not specifically focused on athletic populations were also deprioritized unless they were foundational or directly relevant to diagnosis, imaging, pathoanatomy, or treatment decision-making. Reference lists of selected articles were also reviewed to identify additional relevant studies.

Because this was a narrative review, no formal systematic review protocol, PRISMA flow diagram, or formal risk-of-bias assessment tool was applied. The available literature was interpreted with attention to study design, level of evidence, athlete specificity, and methodologic limitations. Findings were synthesized qualitatively in narrative form according to major clinical themes, including epidemiology, mechanisms of injury, pathoanatomy, clinical presentation, physical examination, imaging evaluation, nonoperative management, operative management, and return-to-play considerations. Conclusions should therefore be interpreted in the context of study heterogeneity and the predominance of retrospective and nonrandomized evidence.

Epidemiology

Posterior shoulder instability accounts for a minority of glenohumeral instability cases and is commonly cited as representing approximately 10% of shoulder instability presentations, although the true prevalence is likely underestimated because many patients do not present with a frank dislocation event [1,2]. In athletic populations, posterior instability appears particularly relevant in contact and collision sports, where repetitive posteriorly directed axial loading places substantial stress on the glenohumeral joint [2,3]. Football players, especially linemen, are classically associated with this pattern, but similar mechanisms may also occur in rugby, wrestling, and other sports involving recurrent contact with the arm positioned in front of the body [2,3].

Compared with anterior instability, posterior instability more often presents with pain, weakness, or activity-related dysfunction rather than a dramatic instability event, which may contribute to delayed recognition and may partly explain why the condition is likely underappreciated in epidemiologic estimates [2,4,5]. This distinction is clinically important because the burden of posterior instability in athletic populations may be greater than suggested by simple dislocation-based reporting alone.

Mechanisms of injury

Mechanisms of injury in posterior shoulder instability may be broadly divided into traumatic and repetitive microtraumatic patterns. Traumatic presentations may follow a single high-force event, such as a direct blow, fall, or posteriorly directed load applied to the shoulder [1]. In contrast, many athletes develop symptoms gradually through repeated loading during blocking, tackling, bench pressing, or other push-based activities that reproduce posteriorly directed force across the glenohumeral joint [2,3].

This mechanism is particularly relevant when the arm is positioned in flexion, adduction, and internal rotation, a posture that increases stress on the posterior capsulolabral structures and may contribute over time to symptomatic microinstability, labral injury, or recurrent posterior translation [1,3]. In contact and collision athletes, repeated exposure to this loading environment helps explain why posterior instability often lacks a single memorable inciting event and instead presents with pain rather than a primary complaint of instability [2-5].

Pathoanatomy

Posterior shoulder stability depends on both static and dynamic stabilizers. Static restraints include the posterior labrum, posterior capsule, and posterior band of the inferior glenohumeral ligament, whereas dynamic stability is provided by the rotator cuff and periscapular musculature [1]. In contact and collision athletes, repetitive posteriorly directed loading may progressively challenge both systems during sport-specific contact and push-based activities. 

In many athletes, the dominant soft-tissue pathology involves the posteroinferior capsulolabral complex. Repetitive axial loading can produce capsular attenuation, labral tearing, or both, contributing to a spectrum of pathology ranging from pain-predominant microinstability to more obvious structural lesions and recurrent symptomatic subluxation [1,3]. One characteristic lesion in posterior instability is the Kim lesion, an incomplete and concealed avulsion of the posteroinferior labrum that may be difficult to appreciate clinically or arthroscopically if not specifically suspected [6]. This lesion also provides an important pathoanatomic bridge to the Kim test discussed later in the physical examination section [6,7].

Osseous morphology also appears to play an important role in posterior instability. Primary imaging studies have demonstrated associations between posterior instability and structural factors such as glenoid dysplasia, increased glenoid retroversion, and posterior glenoid bone loss [8-14]. Harper et al. showed that moderate-to-severe glenoid dysplasia was associated with posterior labral tears on MRI, supporting the concept that morphologic variation may lower the threshold for symptomatic posterior instability [6]. Gottschalk et al. further found that glenoid retroversion was more common in patients with posterior instability, suggesting that version is not simply an incidental imaging feature but may have clinical relevance in recurrence and bilateral instability patterns [7].

Recent work has also clarified posterior glenoid bone loss as more than a secondary descriptor. Prospective data from Bedrin et al. demonstrated that recurrent posterior instability events were associated with greater posterior glenoid bone loss, and that greater retroversion was also associated with more substantial bone loss [8]. Dekker et al. localized posterior glenoid defects most commonly to the posteroinferior quadrant, helping explain why posterior bone loss may behave differently from the more familiar anterior instability pattern [12]. These findings suggest that posterior instability should not be viewed solely as a soft-tissue problem, particularly in athletes with recurrent symptoms or failed prior stabilization.

Biomechanical and clinical threshold studies have attempted to define when bone loss becomes clinically meaningful. Nacca et al. and Arner et al. both support the idea that posterior glenoid bone loss may compromise the success of isolated soft-tissue repair, although exact thresholds remain unsettled and should be interpreted cautiously [10,11]. Taken together, current evidence supports viewing posterior shoulder instability as a pathoanatomic spectrum in which soft-tissue injury, dynamic dysfunction, and osseous morphology may all contribute to symptoms, recurrence risk, and treatment selection.

Clinical presentation

History and Symptom Pattern

Posterior shoulder instability often presents less dramatically than anterior instability, which contributes to delayed recognition in athletic populations. Rather than describing a clear dislocation event, many athletes report vague posterior shoulder pain, weakness, loss of power, mechanical symptoms, or a subjective sense of shifting under load [1-3]. In contact and collision settings, symptoms are commonly linked to blocking, tackling, bench pressing, or other sport-specific push-based activities [2,3].

A careful history is especially important because posterior instability may present along a spectrum, ranging from pain-predominant microinstability to recurrent symptomatic subluxation. Some athletes describe an acute traumatic event followed by persistent instability, whereas others report a more gradual onset of pain and dysfunction related to repetitive loading. Football linemen are a classic example of this gradual-onset pattern related to repetitive blocking mechanics [2,3].

Comparative studies help clarify why posterior instability is frequently underrecognized. In a matched cohort analysis of 200 patients, Bernhardson et al. found that pain was the most common presenting complaint in patients with posterior instability (90.7%), whereas only 13.4% identified instability as their primary complaint [15]. In an athlete-focused comparison, Teske et al. similarly reported that athletes with posterior instability most often presented with pain (96%), whereas athletes with anterior instability were more likely to present with instability as the chief complaint (70%) [16]. These findings help explain why posterior instability is less likely to follow the classic pattern of a memorable dislocation event and more likely to be misattributed to nonspecific shoulder pain or overuse symptoms.

Sport-Specific Presentation

Clinically, athletes may describe deep posterior joint pain, pain with resisted pushing, weakness in forward-flexed positions, painful clicking, diminished contact tolerance, or apprehension when the arm is loaded in front of the body [1-3]. These symptoms should be interpreted in the context of sport-specific demands rather than in isolation.

History taking should also clarify whether symptoms are provoked by contact or by activities that recreate the instability mechanism. Recurrent pain during blocking, inability to tolerate bench press-type movements, loss of confidence in contact situations, or persistent symptoms despite standard rehabilitation should raise suspicion for posterior instability [1-3]. In this setting, the clinical challenge is not simply identifying posterior instability as a binary diagnosis, but recognizing when vague pain and performance limitation reflect clinically meaningful posterior instability rather than nonspecific overuse symptoms.

Physical examination

Physical examination should begin with a standard shoulder assessment, including inspection, scapular posture and mechanics, active and passive range of motion, rotator cuff strength, and evaluation for generalized ligamentous laxity. In contact and collision athletes, the goal is not simply to demonstrate posterior translation on passive testing, but to reproduce the athlete’s symptoms in a clinically meaningful way under positions or maneuvers that approximate sport-specific loading [1,3].

The Kim test and jerk test remain the most commonly cited provocative maneuvers for posterior instability. In the original Kim test study, which evaluated 172 painful shoulders, the Kim test demonstrated a sensitivity of 80% and a specificity of 94% for posteroinferior labral lesions, whereas the jerk test demonstrated a sensitivity of 73% and a specificity of 98%; when used together, sensitivity increased to 97% [5]. In that same study, the Kim test was more sensitive for predominantly inferior lesions, whereas the jerk test was more sensitive for predominantly posterior lesions [5]. However, these figures come from an older single-cohort study, and later systematic review evidence has characterized the support for the Kim test and jerk test as weak when used as stand-alone tests [17].

Accordingly, neither test should be presented as definitive in isolation. Instead, they are best understood as complementary components of a broader examination strategy. This is especially relevant given the pathoanatomic discussion above: the Kim lesion is an incomplete and concealed posteroinferior labral avulsion, and the Kim test is most clinically useful when suspicion for posteroinferior pathology already exists [4,5].

In collision athletes, examination findings should be interpreted in context. Reproduction of posterior pain, apprehension, weakness, or a shifting sensation during positions that mimic blocking, tackling, or resisted pushing may be more informative than passive laxity alone [3,17]. The most useful examination is therefore not the one that generates the largest number of positive special tests, but the one that reproduces the athlete’s symptoms while fitting the broader clinical picture of posterior instability.

Imaging evaluation

Imaging serves as an adjunct to history and physical examination rather than a stand-alone diagnostic tool. Posterior shoulder instability remains primarily a clinical diagnosis, with radiographs and cross-sectional imaging helping identify associated pathology, define predisposing anatomy, and guide treatment planning [18].

Initial evaluation should begin with plain radiographs, including anteroposterior, scapular Y, and axillary views. The axillary view is especially important because posterior humeral head translation, glenoid morphology, and certain bony lesions may not be appreciated adequately on anteroposterior imaging alone [10,18]. In athletes with suspected posterior instability, these views are most useful for screening for gross malalignment, associated osseous injury, and anatomic features that may justify more advanced imaging.

MRI, with or without arthrography, is the primary modality for evaluating soft-tissue pathology. MRI may demonstrate posterior labral tears, capsular injury, chondral damage, reverse Bankart-type lesions, and other intra-articular abnormalities that help explain symptomatic instability [1,18]. However, posterior capsulolabral abnormalities can be subtle, and MRI findings should not be interpreted in isolation because they do not by themselves establish clinically meaningful instability [10,18]. Their value is greatest when they correlate with the athlete’s history, sport-specific mechanism, and examination findings.

CT, particularly with three-dimensional reconstruction, is most valuable when osseous morphology is a major concern. CT can better characterize posterior glenoid bone loss, glenoid retroversion, dysplasia, and humeral-sided bony defects, and it is especially useful in recurrent instability, failed prior stabilization, or cases in which structural bone deficiency may alter operative planning [8,10,12,18]. In this setting, CT adds information that may not be fully captured on MRI alone and is often central to preoperative assessment when bone loss or abnormal morphology is suspected.

Nonoperative management

Nonoperative treatment remains an appropriate initial strategy for many patients with posterior shoulder instability, particularly those with pain-predominant symptoms, atraumatic or microtraumatic instability, and limited osseous pathology. However, this recommendation becomes more nuanced in collision athletes because the shoulder is repeatedly exposed to the same loading pattern that contributed to instability [1,3].

The foundation of nonoperative treatment is structured rehabilitation rather than simple rest. Rehabilitation targets improved dynamic glenohumeral stability through rotator cuff strengthening, scapular stabilization, proprioceptive training, and correction of kinetic chain deficits. The goal is to improve humeral head control under load and reduce functional instability during athletic activity [17,19]. The available rehabilitation literature, however, remains limited, and smaller nonoperative studies should be interpreted cautiously. For example, Blacknall et al. reported patient-reported improvement after a physiotherapy program for atraumatic posterior shoulder subluxation, but the study was small, single-center, and not focused on collision athletes [19].

Available outcomes literature suggests that nonoperative management can succeed in selected patients, but results are mixed and residual symptoms are common. In a population-based retrospective cohort study, Woodmass et al. included 143 patients with posterior shoulder instability, of whom 79 were managed nonoperatively for at least one year after diagnosis. In that subgroup, survival free of surgery after the first year was 78.3% at 1 year, 63.1% at 5 years, and 51.5% at 10 years [20]. These estimates are useful for context but should be interpreted as single-study data rather than definitive benchmarks, particularly when extrapolating to contact and collision athletes [20].

For collision athletes, activity modification is often a limiting factor. Provocative maneuvers such as blocking, bench pressing, push-based lifting, and repeated contact exposure may need to be reduced while rehabilitation progresses, but many athletes cannot avoid these positions long enough to allow symptom resolution [1,3]. This is one reason nonoperative treatment may be less durable in higher-demand populations.

Persistent symptoms, recurrent subluxation, and failure to tolerate athletic demands are therefore reasonable factors favoring surgical management [1].

Operative management

Operative treatment is generally considered for athletes with persistent symptoms despite an appropriate trial of rehabilitation, recurrent symptomatic subluxation, or structural pathology that makes nonoperative management less likely to succeed. In contact and collision athletes, the threshold for surgery is often influenced by sport-specific demands because the same forces encountered during blocking, tackling, or resisted pushing can repeatedly reproduce instability under load [1-3].

For most athletes without substantial posterior glenoid bone loss, arthroscopic posterior stabilization remains the standard operative approach. This typically involves repair of the posterior capsulolabral complex with suture anchors, often combined with capsular plication when capsular laxity is present [13,14]. Available clinical series suggest that arthroscopic stabilization can provide favorable outcomes in appropriately selected patients, but these results should still be interpreted in the context of study design, patient selection, and variability in morphology and athletic demands [13,14].

Recent literature supports generally favorable postoperative outcomes and return-to-sport rates after arthroscopic treatment of posterior instability [13,14,21]. At the same time, these studies do not suggest that all athletes have equivalent risk profiles. In the long-term cohort reported by Rothrauff et al., factors associated with revision included acute postoperative injury, smaller glenoid bone width on MRI, surgery on the dominant extremity, female sex, and repairs performed with fewer than four anchors or anchorless techniques [14]. These findings are clinically useful, but they should be interpreted as associations from cohort-level data rather than as universally generalizable predictors that can be weighted equally in every athlete.

Not all posterior instability is best treated with isolated soft-tissue repair. One of the most important developments in the current literature is the growing recognition that posterior glenoid bone loss and adverse glenoid morphology may affect surgical success [8-11]. Biomechanical and clinical studies have helped define proposed critical bone-loss thresholds, but these thresholds remain less established than those used in anterior instability and should be interpreted cautiously [9-11]. In practice, bone loss is best considered within a broader context that includes glenoid version, dysplasia, recurrence history, and prior stabilization failure.

In selected athletes with substantial posterior glenoid bone loss, failed prior stabilization, significant retroversion, or persistent instability with adverse osseous morphology, bone augmentation or reconstructive procedures may be considered [8,10-12,22]. These procedures are not first-line options for most athletes, but they become more relevant when structural deficiency is likely to compromise the durability of isolated capsulolabral repair.

Return to play

Return-to-Play Principles

Return-to-play decisions should be individualized and based on recovery of function rather than time alone. This is especially important in contact and collision athletes because improvement in the training room does not necessarily mean the shoulder can tolerate the forces encountered during blocking, tackling, or resisted pushing [3]. In practice, return to play should integrate symptom resolution, restoration of sport-specific shoulder function, psychological readiness, and the athlete’s ability to tolerate progressive loading without recurrent instability symptoms [23,24].

Across the available literature, the most consistent return-to-play themes are restoration of pain-free or near pain-free range of motion, near-symmetric strength, recovery of scapular control and proprioception, and successful completion of sport-specific activity without recurrent pain, apprehension, or instability. Systematic review evidence suggests that return-to-sport rates after surgical treatment of posterior instability are generally favorable, but return to the same pre-injury level is more variable and published clearance criteria remain inconsistent [21].

Objective outcome assessment may improve the structure of return-to-play decision-making. The Western Ontario Shoulder Instability Index (WOSI) is a validated patient-reported outcome measure for patients with shoulder instability and remains useful for tracking instability-related quality of life over time [25]. The Posterior Shoulder Instability Questionnaire (PSI-Q) was developed specifically for posterior shoulder instability and demonstrated validity, reliability, and responsiveness, making it a useful tool for monitoring posterior-instability-specific symptoms and function over time [26].

Psychological readiness should also be considered, especially when the athlete reports loss of confidence or fear of recurrent instability despite acceptable strength and motion. The Shoulder Instability-Return to Sport after Injury (SIRSI) scale was developed to assess psychological readiness after shoulder instability and has been shown to identify athletes who are ready to return to the same sport after instability events [23]. More recent work also suggests that SIRSI scores are associated with postoperative outcomes and recurrence after return to sport [27]. In broader shoulder-instability literature, fear of reinjury is one of the most commonly reported impediments to successful return to sport, which supports including psychological readiness in the overall decision-making framework even when posterior-instability-specific data remain limited [24].

Return to Play After Treatment

After nonoperative treatment, return to play depends on whether symptoms truly resolve under athletic loading. Some athletes with pain-predominant or microtraumatic instability may return after focused rehabilitation, but collision athletes are particularly challenging because the underlying mechanism of injury is often reproduced during normal participation [1,3,19]. As a result, progression should be based not only on symptom improvement during standard rehabilitation tasks, but also on tolerance of resisted pushing, contact simulation, and position-specific demands.

After surgical stabilization, return-to-play decisions should likewise be criterion-based rather than purely time-based. In addition to recovery of motion and strength, final clearance should consider dynamic control, restoration of confidence, absence of recurrent instability symptoms, and the athlete’s ability to complete sport-specific progression without meaningful pain or apprehension. For contact and collision athletes, this should ideally include progressive exposure to resisted pushing, contact mechanics, and positional drills before unrestricted return. Because published return-to-play criteria after posterior shoulder stabilization remain variable, clinicians should be cautious about relying on a single timeline in isolation [21].

Because posterior shoulder instability in contact and collision athletes often requires integration of history, examination, and imaging findings rather than reliance on a single feature, the key clinical findings that raise suspicion for posterior instability are summarized in Table 1.

**Table 1:** Clinical presentation, examination, and imaging features that raise suspicion for posterior shoulder instability in contact and collision athletes. Summary of history, symptom pattern, physical examination findings, imaging features, and structural risk factors that may help identify posterior shoulder instability in contact and collision athletes.

Domain	Key findings that raise suspicion for posterior shoulder instability	Clinical relevance
History	Posterior shoulder pain, weakness, loss of power, vague shifting, and symptoms during blocking, tackling, bench pressing, or other push-based activities	Posterior instability often presents without a classic dislocation event and may be underrecognized.
Symptom pattern	Pain-predominant microinstability, gradual onset dysfunction, or recurrent symptomatic subluxation	Helps distinguish subtle presentations from more obvious instability
Sport/mechanism	Repetitive posteriorly directed axial loading with the arm in flexion, adduction, and internal rotation	Suggests a posterior instability mechanism that is common in contact and collision athletes
Physical examination	Kim test, jerk test, and reproduction of posterior pain, apprehension, weakness, or shifting during resisted pushing or sport-specific positions	No single test is definitive; examination findings should be interpreted together and in context
Imaging	Axillary radiograph, MRI for capsulolabral and chondral pathology, and CT for bone loss, retroversion, or dysplasia	Imaging supports the clinical diagnosis and helps define structural pathology relevant to treatment planning.
Structural risk factors	Posterior glenoid bone loss, glenoid retroversion, and glenoid dysplasia	May influence recurrence risk, durability of isolated soft-tissue repair, and the need for more extensive reconstruction

Current controversies and evidence gaps

Despite growing clinical interest in posterior shoulder instability, the evidence base remains less mature than that for anterior instability. Much of the current literature consists of retrospective series, biomechanical studies, review articles, and recent expert consensus statements rather than high-level prospective comparative trials [1,2]. In addition, important sources of heterogeneity persist across studies, including differences in traumatic versus microtraumatic presentations, contact versus non-contact athletes, primary versus revision cases, and the presence or absence of glenoid dysplasia, retroversion, or bone loss. These differences complicate direct comparison across cohorts and limit the strength of generalized treatment recommendations [8,11].

One of the most important unresolved issues is how osseous morphology should influence treatment selection. Current evidence supports the view that posterior glenoid bone loss, glenoid retroversion, and dysplasia are clinically relevant rather than incidental findings [8,10,11]. However, unlike anterior instability, posterior instability still lacks universally accepted structural thresholds that clearly dictate when isolated soft-tissue repair is insufficient and when augmentation or reconstruction should be preferred. Existing biomechanical and clinical studies are informative, but they do not yet provide a single decision rule that can be applied consistently across athletic populations [9-11]. As a result, treatment decisions still require individualized interpretation of bone loss, version, recurrence history, sport-specific demands, and prior surgical history.

Another unresolved area is the transition from rehabilitation to surgery and, later, from recovery to return to play. In theory, many athletes with pain-predominant or microtraumatic posterior instability deserve an initial trial of structured rehabilitation [1,20]. In practice, this may be less predictable in contact and collision athletes because symptom-provoking sport demands are often difficult to avoid [1,3,20]. Similarly, although return-to-sport rates after surgery are generally favorable, the criteria used to clear athletes remain variable across studies [21]. More recent work has improved this framework by incorporating validated outcome tools and psychological readiness measures, but a standardized posterior-instability-specific return-to-play model has not yet been established [23-27].

Taken together, these gaps suggest that future work should move beyond simply reporting additional case series and instead focus on prospective athlete-specific studies, clearer stratification by morphology and instability pattern, and more standardized treatment and return-to-play criteria. That type of work would be more likely to improve clinical decision-making than repeating broad outcome summaries alone.

Clinical practice points

Posterior shoulder instability in contact and collision athletes often presents with pain, weakness, or performance limitation rather than a clear dislocation event, so delayed recognition is common and diagnosis requires a high index of suspicion.

Evaluation should emphasize correlation of history, physical examination, and imaging findings rather than reliance on any single provocative maneuver or isolated imaging abnormality.

Posterior instability is best understood as a clinical spectrum, ranging from pain-predominant microinstability to recurrent symptomatic subluxation associated with capsulolabral injury and, in some athletes, clinically meaningful osseous pathology.

The Kim test and jerk test are best used as complementary examination maneuvers within a broader clinical assessment and should not be interpreted as definitive in isolation.

Nonoperative treatment remains an appropriate initial strategy in selected athletes, but collision athletes may be less predictable candidates for prolonged rehabilitation because the provoking mechanism is often difficult to avoid during participation.

Treatment planning should account for structural morphology, including posterior glenoid bone loss, retroversion, and dysplasia, because these factors may influence the durability of isolated soft-tissue repair and the need for more extensive reconstruction.

Return-to-play decisions should be individualized and criterion-based, incorporating pain-free function, near-symmetric strength, dynamic control, psychological readiness, and tolerance of sport-specific loading rather than time alone.

## Conclusions

Posterior shoulder instability in contact and collision athletes is an underrecognized but clinically important cause of shoulder pain, dysfunction, and impaired athletic performance. Unlike anterior instability, it often presents without a dramatic instability event and instead requires careful pattern recognition based on history, examination, and imaging interpreted in the proper clinical context. Current evidence supports viewing posterior instability as a spectrum rather than a single entity. In some athletes, symptoms are driven primarily by pain-predominant microinstability and repetitive loading; in others, recurrent symptomatic subluxation, capsulolabral injury, and clinically relevant osseous morphology play a more prominent role. This distinction matters because successful management depends on matching treatment to both the athlete’s structural pathology and the functional demands of the sport.

Nonoperative treatment remains an appropriate starting point for selected athletes, but collision athletes may be less predictable candidates for prolonged rehabilitation because the same loading patterns that provoke symptoms are often unavoidable during participation. Arthroscopic stabilization generally provides favorable outcomes in properly selected patients, although posterior glenoid bone loss, dysplasia, retroversion, and prior instability history may influence the durability of soft-tissue repair and should be considered carefully during treatment planning. Return-to-play decisions should be individualized and criterion-based rather than determined by time alone. Recovery of pain-free function, near-symmetric strength, dynamic control, psychological readiness, and tolerance of sport-specific loading all remain central to safe return. As the evidence base continues to evolve, future progress will depend less on repeating broad outcome summaries and more on athlete-specific prospective studies, clearer morphologic stratification, and more standardized treatment and return-to-play frameworks.
